# CD148 agonistic antibody alleviates renal injury induced by chronic angiotensin II infusion in mice

**DOI:** 10.1186/s12882-025-04070-x

**Published:** 2025-03-31

**Authors:** Keiko Takahashi, Alina Yu, Tadashi Otsuka, Lejla Pasic, Chikage Narui, Lilly He, Philipp Ellinger, Manuel Grundmann, Raymond C. Harris, Takamune Takahashi

**Affiliations:** 1 Division of Nephrology and Hypertension, Vanderbilt University Medical Center, S-3223 Medical Center North, 1161 21st Avenue South, Nashville, TN 37232 USA; 2 Department of Biochemistry, Vanderbilt University, Nashville, TN 37232 USA; 3 Bayer AG Research & Development, Pharmaceuticals, 42113 Wuppertal, Germany

**Keywords:** Protein tyrosine phosphatase, CD148, Agonistic antibody, Angiotensin II, Epidermal growth factor receptor, Hypertensive nephropathy, Renal fibrosis

## Abstract

**Background:**

Angiotensin II (Ang II) plays a critical role in the progression of kidney disease. In addition to its direct signaling events, Ang II transactivates epidermal growth factor receptor (EGFR) and causes renal injury. CD148 is a transmembrane protein tyrosine phosphatase that dephosphorylates EGFR and strongly inhibits its activity. In this study, we have asked if CD148 agonistic antibody 18E1 mAb attenuates renal injury induced by chronic Ang II infusion to explore its therapeutic application.

**Methods:**

Hypertensive nephropathy was induced in mice subjected to unilateral nephrectomy (UNx) by infusing Ang II (1.4 mg/kg per day) for 6 weeks using an osmotic minipump. The 18E1 mAb or isotype control IgG were intraperitoneally injected (15 mg/kg, three times per week) to the UNx + Ang II mice for 6 weeks, and their renal phenotype was investigated.

**Results:**

Chronic Ang II infusion induced evident hypertension and renal injury that is indicated by elevation of plasma creatinine, urinary albumin excretion, renal hypertrophy, podocyte injury, macrophage infiltration, and the expression of alpha smooth muscle actin and collagen deposition. As compared with isotype control antibody, 18E1 mAb significantly reduced these renal changes, while it showed no effects on blood pressure. Furthermore, phospho-EGFR immunohistochemistry and immunoblotting demonstrated renal EGFR is activated in the mice that were subjected to UNx and Ang II infusion and 18E1 mAb significantly reduces EGFR phosphorylation in these kidneys as compared with isotype control treatment.

**Conclusion:**

Agonistic CD148 antibody attenuates UNx + Ang II–induced renal injury, in part by reducing EGFR activity.

**Supplementary information:**

The online version contains supplementary material available at 10.1186/s12882-025-04070-x.

## Background

Clinical and experimental studies have shown that angiotensin II (Ang II), the major effector of renin-angiotensin system (RAS), plays a critical role in the progression of various kidney diseases including hypertensive nephropathy and diabetic nephropathy [1]. Elevated renal expression and action of Ang II are major factors involved in the initiation and progression of chronic kidney disease (CKD) [1], causing renal injury through renal hemodynamic effects and stimulation of kidney growth, phenotypic change, inflammation and matrix deposition [1–3]. RAS inhibitors, including angiotensin-converting enzyme inhibitors and Ang II receptor type 1 (AT1) blockers, attenuate renal injury in experimental models and slow progressive loss of renal function in patients with CKD [4, 5].

Ang II transduces the signals through at least two distinct G protein-coupled receptors, AT1 and AT2 [6]. Renal cells, including tubules as well as vascular cells, predominantly express AT1 that mediates the majority of known Ang II actions [7, 8]. In addition to direct intracellular signaling events, AT1 activation can lead to transactivation of epidermal growth factor receptor (EGFR) by either an EGFR ligand-dependent or -independent mechanisms [9–12]. EGFR and its ligands are abundantly expressed along the nephron, and importantly a body of literature has shown that EGFR activation causes progressive renal injury [13, 14] and that EGFR mediates the detrimental effects seen with chronic AT1 activation [12]. Furthermore, aldosterone, the main mineralocorticoid steroid hormone released from the adrenal gland by Ang II, was shown to activate EGFR through the production of reactive oxygen species, and erlotinib, a small molecule EGFR inhibitor, effectively suppresses renal structural changes and fibrotic responses induced by aldosterone infusion [15, 16]. These findings indicate EGFR activation plays a key role in renal injury caused by activation of RAS and EGFR inhibition is a promising therapeutic approach for progressive kidney disease. However, the toxicity of EGFR inhibitors, especially skin toxicity, hampers their use for kidney disease [17].

CD148 is a transmembrane protein tyrosine phosphatase (PTP) that is expressed in various cell types and organs, including renal vasculature and tubules [18]. Previous studies have shown that CD148 negatively regulates growth factor signals by interacting with- and dephosphorylating growth factor receptors and their signaling molecules, inhibiting cell proliferation and transformation. These include EGFR [19–23], vascular endothelial growth factor receptor-2 (VEGFR-2) [21, 24, 25], platelet-derived growth factor receptor-β (PDGFRβ) [26, 27], ERK1/2 [28, 29], PLCγ1 [30, 31], and p85 subunit of phosphoinositide 3-kinase [32]. Of note, an unbiased screen has identified CD148 as a PTP that strongly inhibits EGFR signaling [19], and this activity was confirmed by subsequent studies [20–23]. We have recently developed agonistic CD148 monoclonal antibody 18E1 mAb and showed that the antibody attenuates diabetic nephropathy in streptozotocin-induced diabetic mice [18]. In the present study, we asked if CD148 agonistic antibody alleviates renal injury induced by chronic Ang II infusion. Our data demonstrates that agonistic CD148 antibody attenuates Ang II–induced renal injury accompanied by reduction of EGFR phosphorylation.

## Methods

## Animals

DBA/2 J strain mice were purchased from The Jackson Laboratory (Bar Harbor, Maine). CD148 LacZ knock-in mice with DBA/2 J strain were generated and genotyped as described previously [18].

## Chronic administration of Ang II and treatment with 18E1 mAb

Ten-week-old DBA/2 J male mice were anesthetized by intraperitoneal injection of 75 mg/kg Ketamine, 0.5 mg/kg Dexmedetomidine mixture and subjected to unilateral nephrectomy (UNx) by removing the right kidney, then Ang II (1.4 mg/kg per day, Sigma-Aldrich. St. Louis, MO) was infused for 6 weeks using an osmotic minipump (Alzet MICRO-OSMOTIC PUMP MODEL 1002, Alza Corporation, Palo Alto, CA). The 18E1 mAb was generated by immunizing CD148 knockout mice and its agonistic activity was evaluated by cell-based and biochemical assays [18]. The 18E1 mAb or isotype control IgG were produced and intraperitoneally injected to the mice (15 mg/kg, three times per week) for 6 weeks as described previously [18]. Mice with sham operation were used as a control. Urine collection, blood sampling, and blood pressure measurements were performed before and at 4 and 6 weeks after Ang II infusion. At the end of study, mice were euthanized, and blood (via the left ventricle) and kidneys were sampled for analysis. Euthanasia was carried out by inhalation of carbon dioxide overdose and subsequent cervical dislocation.

## Measurements of blood pressure, blood glucose, plasma creatinine, and urinary albumin excretion

Systolic blood pressure (SBP), fasting blood glucose, and urinary albumin to creatinine ratio (ACR) were measured as described previously [18]. In brief, blood was sampled by saphenous vein puncture after a 6-h fasting, and blood glucose was measured using Accu-Chek test strips (Roche Applied Science, Indianapolis, IN). Systolic blood pressure was measured in conscious trained mice at room temperature using a tail-cuff monitor (BP-2000 Series II Blood Pressure Analysis System, Visitech Systems, Apex, NC). Urinary albumin excretion was assessed by the determination of albumin-to-creatinine ratio (ACR) of 24-h urine. Twenty four-hour urine was collected from individually caged mice using polycarbonate metabolic cages (Tecniplast, Buguggiatte, Italy). Urinary albumin and creatinine were measured using the Albuwell M Microalbuminuria ELISA kit (Exocell Inc., Philadelphia, PA) and the Creatinine Companion kit (Exocell Inc.), respectively. Plasma creatinine was determined by underivatized stable isotope dilution LC-MS/MS [33] at UAB O’Brien Center (Birmingham, AL).

## Histological, histochemical, and immunohistochemical assessment of renal phenotype

Periodic Acid-Schiff stain and β-galactosidase histochemistry were carried out as described previously [18] and photographed by light microscopy (Olympus BX53, Olympus Life Sciences, Tokyo, Japan). Podocyte number and macrophage infiltration were assessed by Wilms’ tumor gene 1 (WT1) and F4/80 immunohistochemistry (IHC) and quantified as described [18, 34]. In brief, podocyte number was assessed by counting WT1-positive cells in glomerular cross section. At least 20 cortical glomeruli were assessed in each mouse. Macrophage infiltration was assessed by measuring F4/80-immunostained cells with a fixed color threshold. At least 20 high-power fields (x400) that contain cortical glomeruli were assessed as macrophage infiltration was abundant in periglomerular and interstitial regions. Results were expressed as the percentages in the area. Renal fibrosis was assessed by alpha smooth muscle actin (αSMA) IHC and picrosirius red stain and quantified as described previously [35]. For αSMA IHC, 4% paraformaldehyde fixed paraffin sections (4 µm) were antigen retrieved in 0.01 M citrate buffer (pH 6.0, Sigma Aldrich) for 15 min using a pressure cooker. Sections were cooled for 30 min at room temperature (RT) and blocked in 0.3% H_2_O_2_ in methanol followed by 1x Universal Blocking Reagent (BioGenex, Fremont, CA) for 30 min. The sections were then incubated with horseradish peroxidase-conjugated αSMA antibody (Abcam, Inc., Cambridge, United Kingdom) for overnight at 4 °C. Immunoreactions were visualized using the Peroxidase DAB Substrate Kit (Vector Laboratories, Burlingame, CA). Dehydrated tissue sections were mounted (Cytoseal XYL, Thermo Fisher Scientific, Grand Island, NY) and photographed by light microscopy (Olympus BX53). Picrosirius red stain was carried out as described previously [35]. The αSMA immunoreactivity and picrosirius red-stained area were quantified as described [35]. The phospho-EGFR (p-EGFR) IHC was carried out and quantified as follows. Four percent paraformaldehyde-fixed paraffin sections were dewaxed, rehydrated, then heated in TE pH 9.0 buffer via a pressure cooker for 15 min, then cooled for at least 30 min to RT. Sections were blocked in 0.3% H_2_O_2_ in methanol followed by the Avidin/Biotin Blocking Kit (Vector Laboratories) and 1x Universal Blocking Reagent (BioGenex). The sections were incubated with rabbit phospho-specific EGFR Y1068 antibody (D7A5, Cell Signaling Technology, Danvers, MA) that detects activated EGFR overnight at 4 °C, washed, then incubated with biotinylated anti-rabbit IgG (Vector Laboratories) for 1 hr at RT. Immunoreactions were detected using the VECTASTAIN Elite ABC HRP Kit (Vector Laboratories) and Peroxidase DAB Substrate Kit (Vector Laboratories). Tissues were dehydrated, mounted with Cytoseal XYL (Thermo Fisher Scientific), and imaged at 20x magnification to a resolution of 0.5 µm/pixel using a high throughput Leica SCN40 Slide Scanner (Leica Biosystems, Deer Park, IL). Intensity of p-EGFR immunostaining was quantified using QuPath version 0.4.3 [36] as follows. Tissue images were annotated to isolate the cortex area. A pixel thresholder was created to detect positive vs. negative p-EGFR staining. Sections that were immunostained without a primary antibody were considered as negative p-EGFR staining. The adjusted thresholder was then run across the image annotations to measure p-EGFR positive area in renal cortex.

## Immunoblotting

Kidney cortex tissues were homogenized and lysed in NP-40 lysis buffer [10 mM Tris-HCl (pH 8.0), 150 mM NaCl, 10% glycerol, 1% NP-40, 2 mM sodium vanadate, 5 mM EDTA, 5 mM NaF, cOmplete Protease Inhibitor Cocktail and PhosSTOP (Roche)]. Lysates were clarified by centrifugation at 14,000 rpm for 10 min at 4 °C, and total protein concentration was measured using the Pierce BCA Protein Assay Kit (Thermo Fisher Scientific). Equal protein amounts (250 µg) were mixed with NuPAGE 4x LDS Sample Buffer and 10x Sample Reducing Agent (Thermo Fisher Scientific), boiled, separated on 7.5% Criterion™ TGX™ Precast Midi Gels, then transferred onto nitrocellulose membranes using the Trans-Blot Turbo Transfer System (Bio-Rad, Hercules, CA). Membranes were blocked with Intercept (TBS) Blocking Buffer (LI-COR Biosciences, Lincoln, NE) for 1 hr at RT prior to overnight primary antibody incubation at 4 °C. Primary antibodies included phospho-EGFR Y1068 (D7A5, Cell Signaling Technology) and total EGFR (E1282, Sigma-Aldrich) antibodies. Membranes were washed and incubated with LI-COR IRDye® secondary antibodies for 1 hr at RT and imaged with the Odyssey CLx Imager (LI-COR Biosciences). Band intensities were quantified with the LI-COR Image Studio software (LI-COR Biosciences), and phospho- to total EGFR ratios were calculated.

## Statistical analysis

Data are expressed as means ± SD. Statistical analysis was performed using Prism 10 (GraphPad Software Inc., La Jolla, CA). Differences between the experimental groups were evaluated using one-way analysis of variance with post-hoc Tukey honestly significant difference test. *P* < 0.05 was considered as statistically significant.

## Results

## Chronic Ang II infusion induces CD148 expression in renal tubules

**Fig. 1:**
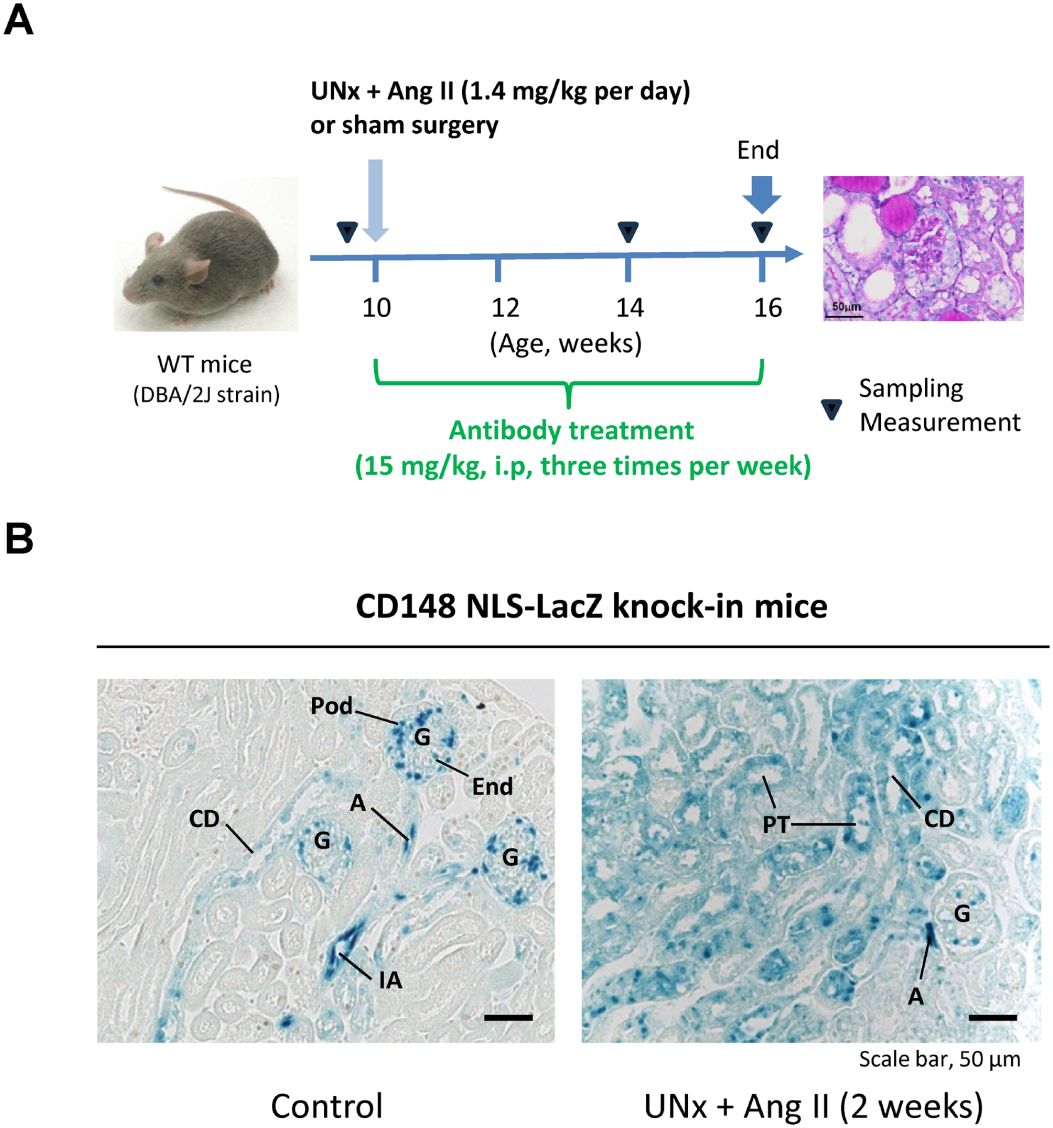
Experimental protocol and CD148 expression in Ang II–infused mouse kidney. (**A**) Ten-week old male DBA/2 J strain mice were subjected to unilateral nephrectomy (UNx), then osmotic minipumps were implanted subcutaneously, infusing Ang II (1.4 mg/kg/day) for 6 weeks. 18E1 mAb (n = 7) or isotype control (n = 7) was intraperitoneally injected to the mice (15 mg/kg, three times per week) for total 6 weeks. The DBA/2 J mice (n = 7) that received sham operation (no UNx, no infusion) were used as a control. Measurements or sampling were performed before and at 4 and 6 weeks after Ang II infusion. (**B**) CD148 NLS-LacZ knock-in mice (DBA/2 J strain) were subjected to UNx + Ang II infusion (2 weeks) (n = 4) or sham operation (control, n = 3). CD148 promoter activity was assessed in these kidneys by β-galactosidase histochemistry. Representative data is shown. *G* glomerulus; *IA* interlobular artery; *CD* collecting duct; *Pod* podocyte; *End* glomerular endothelial cell; *A* arteriole; *PT*, proximal tubule

In the present study, we examined the effects of CD148 agonistic antibody 18E1 mAb [18] in renal injury induced by chronic Ang II infusion. Chronic Ang II infusion was carried out for 6 weeks using an osmotic minipump with DBA/2 J strain mice that were subjected to UNx. The 18E1 mAb or isotype control IgG was intraperitoneally injected to the mice for 6 weeks and renal phenotype was analyzed (Fig. 1A). DBA/2 J mice that were subjected to sham operation were used as a control.

First, we examined CD148 expression in Ang II–infused kidneys using the CD148 LacZ knock-in mice (DBA/2 J strain) [18] and β-galactosidase histochemistry. In this LacZ knock-in mice, the LacZ gene has a nuclear localization sequence (NLS); therefore, β-galactosidase activity is observed in nucleus. As shown in Fig. 1B (left panel), β-galactosidase activity, demonstrating CD148 promoter activity, was observed in arterial endothelia, glomeruli (podocytes, a subpopulation of glomerular endothelial cells) and collecting ducts in CD148 LacZ knock-in mice that were subjected to sham operation. The same pattern of CD148 promoter activity has been shown in intact CD148 LacZ knock-in mice [18]. In contrast, CD148 expression was also observed in tubular segments other than collecting ducts, including proximal tubules, in CD148 LacZ knock-in mice that were subjected to UNx and 2-week Ang II (1.4 mg/kg per day) infusion (Fig. 1B, right panel), indicating that Ang II induces CD148 expression in renal tubules. CD148 LacZ knock-in mice that were subjected to UNx alone showed similar CD148 expression to the mice with sham operation (Supplemental Fig. S1).

## CD148 agonistic antibody alleviates renal injury induced by chronic Ang II infusion

**Fig. 2:**
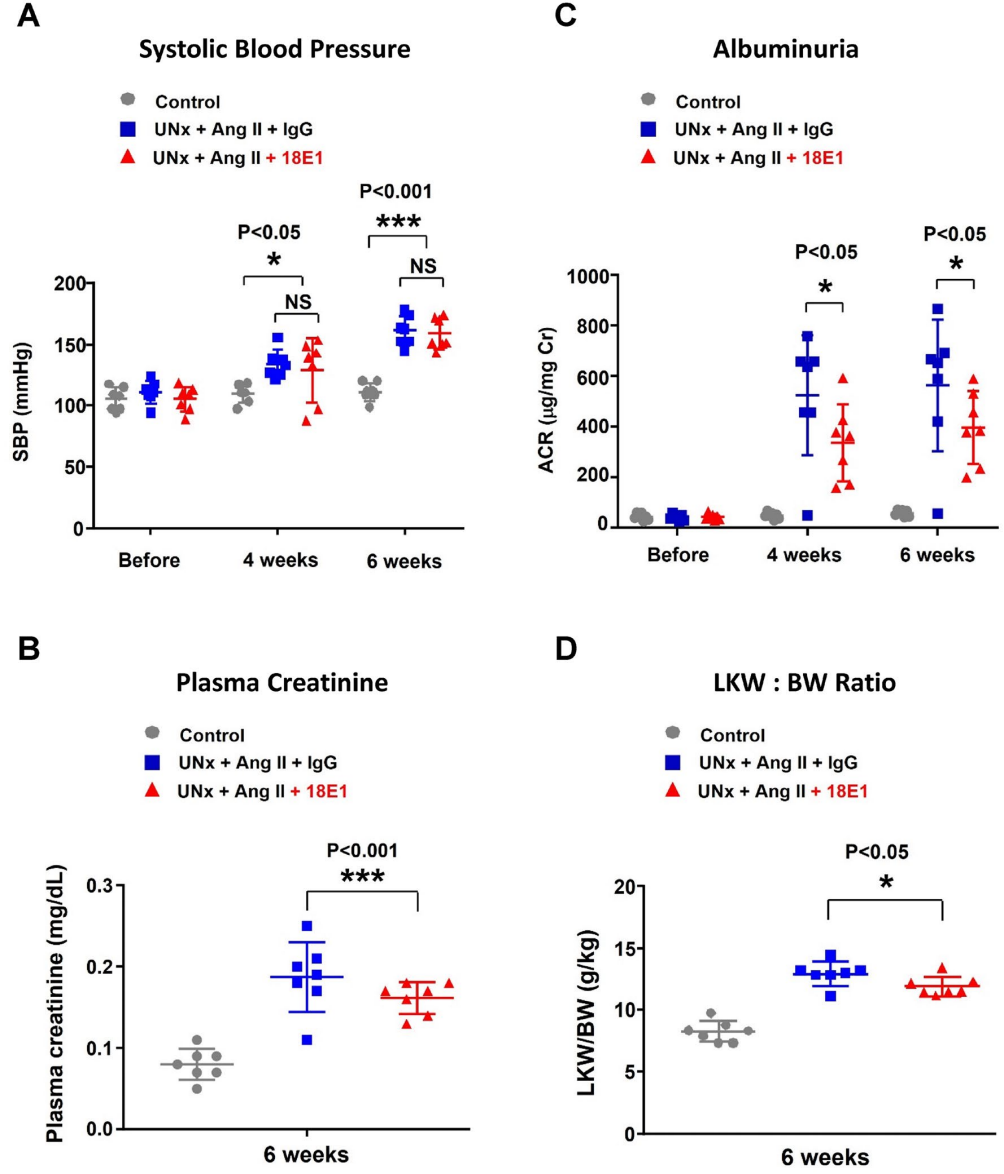
The effects of 18E1 mAb on physiological and renal parameters. Systolic blood pressure (SBP) (**A**), plasma creatinine (**B**), urinary albumin-to-creatinine ratio (ACR) (**C**) and left kidney weight to body weight ratio (LKW/BW) (**D**) were assessed in each group of mice at the indicated time points. Data are presented as means ± SD. n = 7 for each experimental group

Figure 2 shows the results of our phenotypic analysis of each group of mice. Compared to control mice, the mice that were subjected to UNx + Ang II infusion showed elevation of SBP (Fig. 2A) and increases in plasma creatinine (Fig. 2B), urinary albumin excretion (Fig. 2C), and left kidney weight to body weight ratios (LKW/BW) (Fig. 2D). Although no significant difference was observed in SBP between 18E1 mAb- and isotype control IgG-treated UNx + Ang II mice, 18E1 mAb-treated mice showed significantly lower plasma Cr (at 6 weeks), urinary albumin excretion (at 4 and 6 weeks), and LKW/BW ratios (at 6 weeks) (Fig. 2A–D), indicating that 18E1 mAb alleviates renal injury and structural change induced by UNx and Ang II infusion. Although CD148 was shown to reduce insulin signaling when it is co-transfected with insulin receptor in HEK293T cells [37], we could not see the difference in fasting blood glucose level between 18E1 mAb- and isotype control-treated mice (0.114 ± 0.019 vs. 0.116 ± 0.011 mg/dL, *P* = 0.5501, n = 7 per group).

**Fig. 3:**
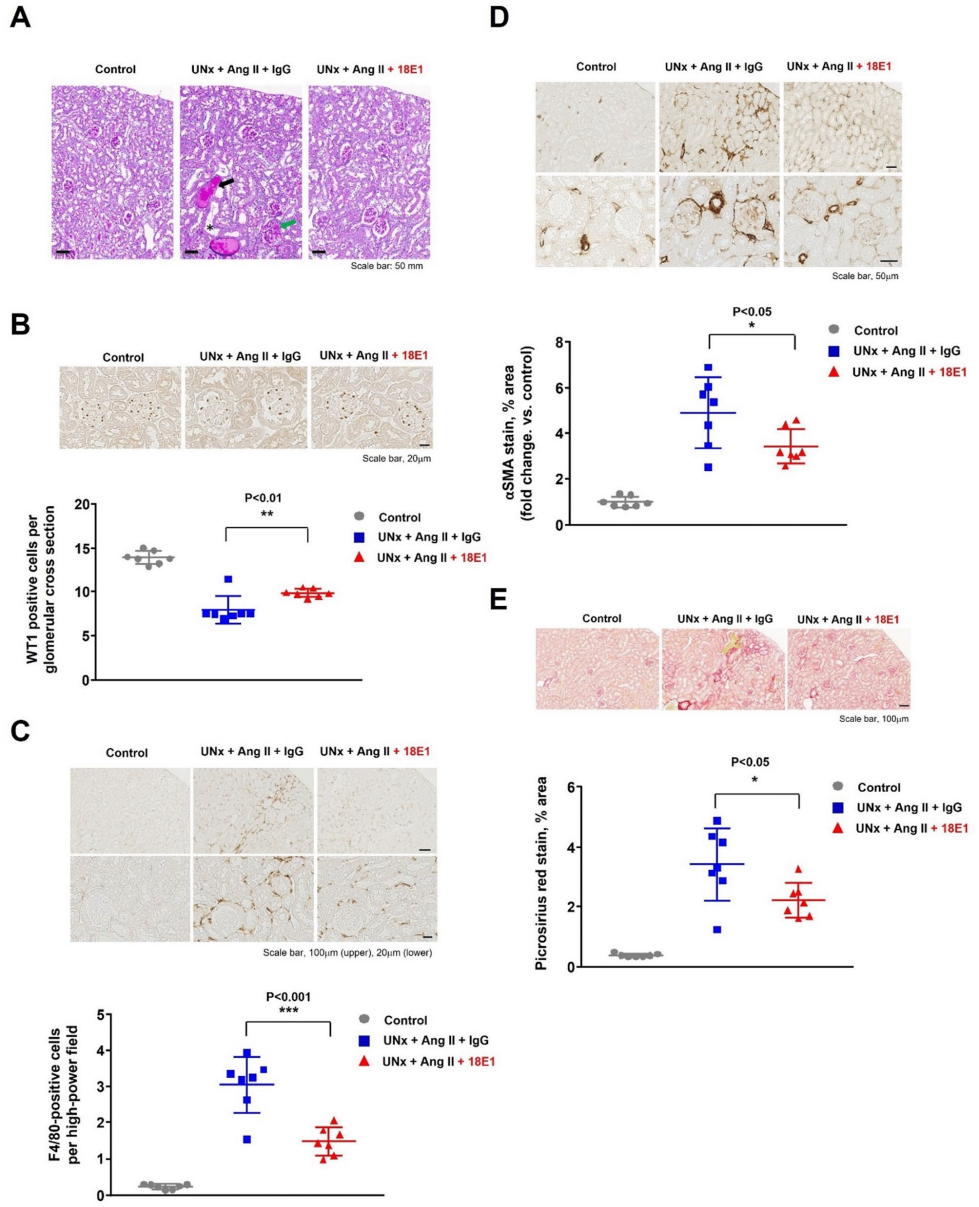
The effects of 18E1 mAb on renal pathology. Renal histopathology was assessed by Periodic Acid-Schiff stain (**A**). Podocyte number in glomerular cross section (**B**) and macrophage infiltration (**C**) were assessed by WT1 and F4/80 IHC respectively. Renal fibrosis was assessed by αSMA IHC (**D**) and picrosirius red stain (**E**). The results were quantified as described in the “Methods”. Photographs show representative results. Data are presented as means ± SD. n = 7 for each experimental group. In panel A, green arrow indicates sclerotic glomerulus, black arrow hyaline cast, and asterisk dilated tubule respectively

We next assessed renal injury in UNx + Ang II mice by histological and immunohistochemical investigation. Mice that were subjected to UNx + Ang II infusion showed renal lesions, including glomerulosclerosis (Fig. 3A), tubular dilatation and atrophy (Fig. 3A), podocyte injury as evidenced by reduced numbers of WT1-positive podocytes in glomerular cross section (Fig. 3B), macrophage infiltration (Fig. 3C), and fibrogenesis or interstitial fibrosis indicated by αSMA expression (Fig. 3D) or collagen depositions (picrosirius red stain) (Fig. 3E). These pathological changes were significantly less in 18E1 mAb-treated mice as compared with isotype control-treated mice (Fig. 3A–E), indicating that 18E1 mAb alleviates renal injury induced by UNx and chronic Ang II infusion. In addition to these studies, we also subjected homozygous CD148 LacZ knock-in mice to UNx and chronic Ang II infusion and treated the mice (n = 5 per group) with either 18E1 mAb or isotype control to confirm the on-target effects of 18E1 mAb. However, these mice were quite sick, showing body weight loss; thus, we had to terminate the study. This finding also suggests that CD148 has activity protecting tissue injuries induced by Ang II infusion.

## CD148 agonistic antibody reduces EGFR activation in Ang II infused mouse kidney

**Fig. 4:**
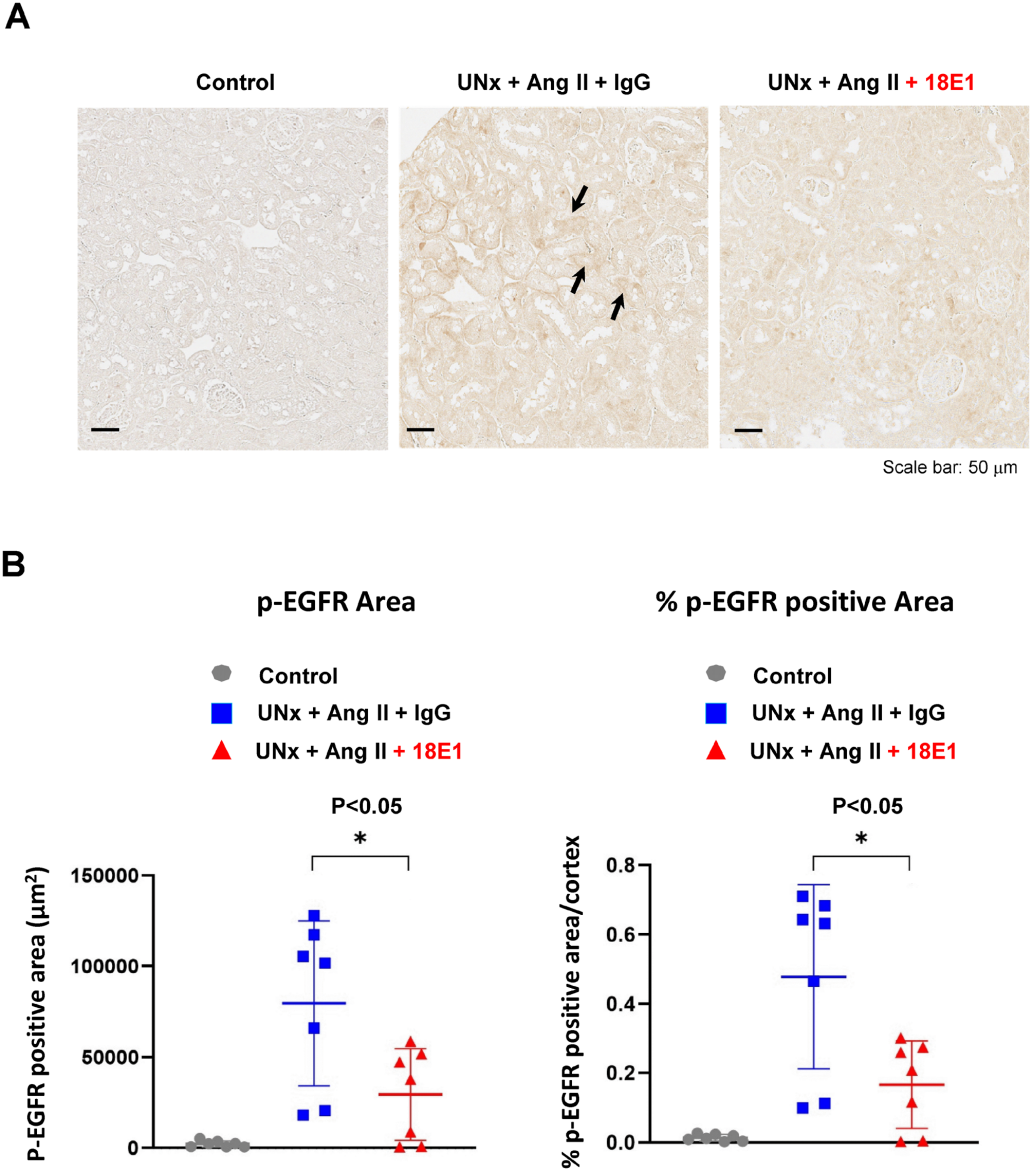
Immunohistochemical assessment of EGFR phosphorylation. EGFR phosphorylation in renal cortex was assessed by IHC using the phospho-specific EGFR (Y1068) antibody (**A**) and quantified (**B**) as described in the “Methods”. Photographs show representative results. Black arrows indicate EGFR phosphorylation in renal tubules. Data are presented as means ± SD. n = 7 for each experimental group

**Fig. 5:**
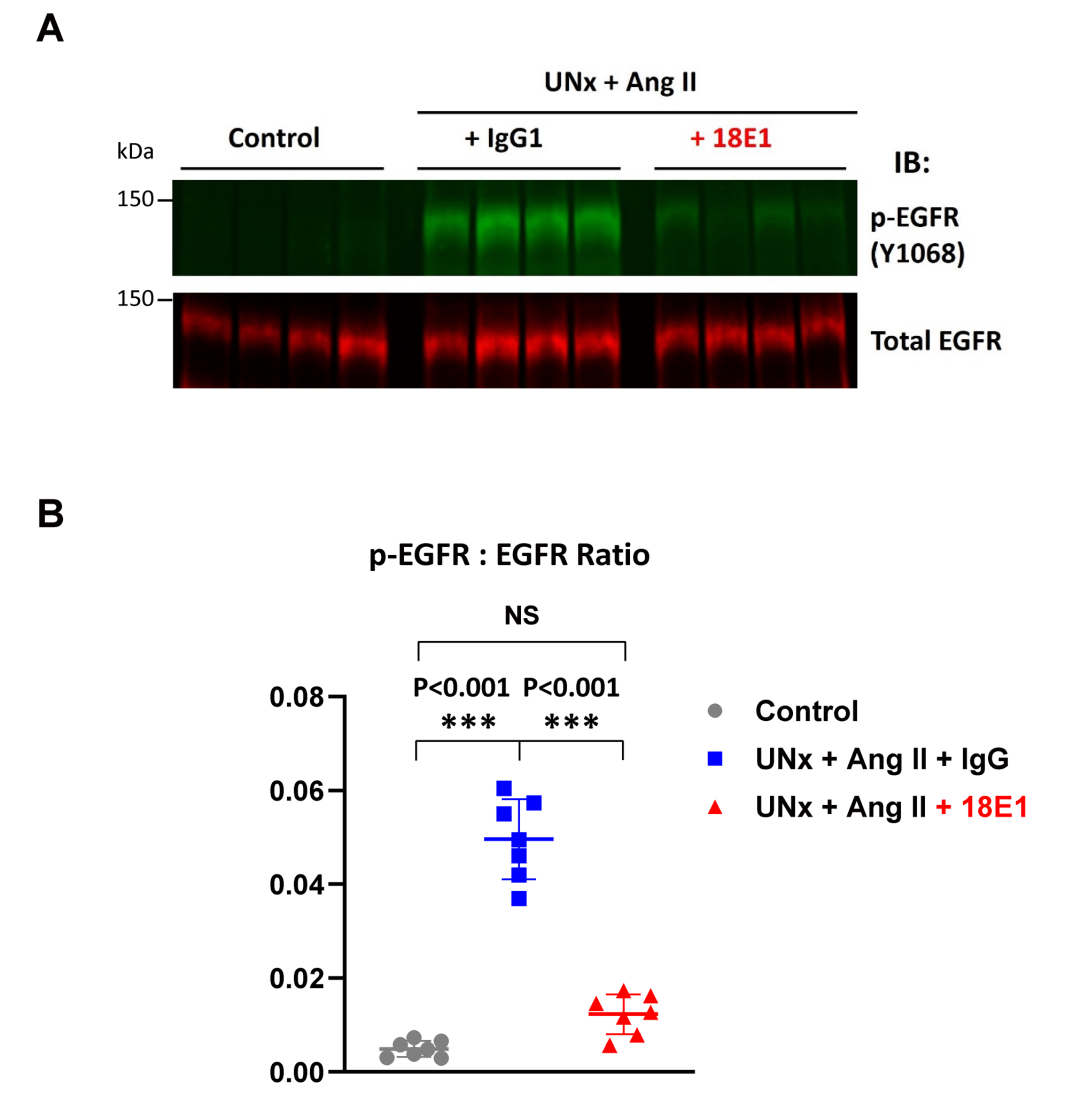
Immunoblot analysis of EGFR phosphorylation. EGFR phosphorylation in renal cortex was assessed by immunoblotting using the phospho-specific (Y1068) and total EGFR antibodies as described in the “Methods”. Representative images (four mice per group) (**A**) and quantification of the ratio of phospho- to total EGFR signal (**B**) are shown. Data are presented as means ± SD. n = 7 for each experimental group. IB, immunoblotting

Last, we assessed the effects of 18E1 mAb on EGFR activation in the kidneys subjected to UNx + Ang II infusion by IHC and immunoblotting with phospho-specific EGFR Y1068 antibody. As shown in Fig. 4A, EGFR Y1068 phosphorylation was highly limited in control kidneys, while kidneys of UNx + Ang II mice showed regional increases in EGFR Y1068 phosphorylation in renal tubules. EGFR phosphorylation was also observed in the podocytes of sclerotic glomeruli in UNx + Ang II mice (Supplemental Fig. S2). Compared to isotype control-treated UNx + Ang II mice, p-EGFR Y1068 immunostaining was weaker in the kidneys of UNx + Ang II mice treated with 18E1 mAb (Fig. 4A). Figure 4B shows quantification of p-EGFR Y1068 immunostaining in each group of mice. Phospho-EGFR Y1068 positive area was significantly reduced in 18E1 mAb-treated mouse kidneys as compared with isotype control-treated kidneys. The results were further confirmed by immunoblotting (Fig. 5). No difference was observed in total EGFR IHC between these two groups (Supplemental Fig. S3). These findings indicate that 18E1 mAb inhibits renal EGFR activation induced by UNx + Ang II infusion.

## Discussion

This work investigated the effects of CD148 agonistic antibody in renal injury induced by chronic Ang II infusion. Our data demonstrates the first evidence that Ang II–induced renal injury can be attenuated by CD148 agonism. Ang II was shown to induce renal hypertrophy, glomerular injury and glomerulosclerosis, tubular injury, atrophy and epithelial-to-mesenchymal transition, interstitial fibrosis, and inflammatory cell infiltration including macrophages [12, 34, 38]. These events were attenuated by CD148 agonistic antibody 18E1 mAb. Given the facts that EGFR activation by Ang II largely contributes to these pathological events [10–12, 34, 38–40] and CD148 strongly inhibits EGFR signaling [19, 20, 22, 23], the observed protective effects of CD148 agonistic antibody would be in part due to the reduction of EGFR activity. Indeed, EGFR phosphorylation was significantly reduced in the kidneys of UNx + Ang II mice treated with 18E1 mAb. Our previous work also demonstrated that 18E1 mAb has the activity in inhibiting EGFR signaling in cultured podocytes [18]. Further study would be required to clarify the causal relationship between 18E1 mAb effects and reduction of EGFR activity, including asking if Wave-2 mice [41], which show reduced EGFR activity, largely attenuate the effects of 18E1 mAb. In addition to inhibition of EGFR signaling, CD148 was shown to suppress several growth factor signals that have been implicated in kidney pathology, including PDGFRβ [26, 27], CSF1R [42, 43] and TGFβR [44, 45]. Therefore, CD148 agonistic antibody could attenuate Ang II–induced renal injury by reducing these growth factor signals as well as EGFR signal. It would also be interesting to compare the efficacy of 18E1 mAb and EGFR inhibitor.

In the present study, CD148 agonistic antibody did not alter blood pressure in UNx + Ang II mice. Several studies have shown a role of EGFR in regulation of blood pressure [46]. Ligands for EGFR, including EGF and HB-EGF, were shown to have an activity to induce vasoconstriction [47–50]. It was also shown that Ang II–induced cardiac hypertrophy and hypertension are attenuated by EGFR antisense oligonucleotide [51]. On the other hand, EGFR is expressed in collecting ducts as well as in vascular cells, and infusion of EGF decreases epithelial sodium channel (ENaC) activity and prevents the development of hypertension and glomerular and tubular damage in Dahl SS rat strain [46, 52]. It was also shown that anti-EGFR monoclonal antibody Cetuximab elevates blood pressure accompanied by reduction of ENaC phosphorylation in mice [53]. CD148 is expressed in both vascular cells and collecting ducts; therefore, it could regulate blood pressure positively and negatively via EGFR. This may be the reason why CD148 agonistic antibody showed no evident effects on blood pressure.

The 18E1 mAb also attenuates renal hypertrophy in UNx + Ang II mice. Since Ang II advances renal hypertrophy in UNx mice and EGFR is involved in this process [10, 34], this could be via EGFR inhibition. However, currently it is unclear that 18E1 mAb attenuates compensatory renal hypertrophy itself. We have demonstrated that UNx alone does not cause EGFR phosphorylation [34]. Furthermore, it was shown that anti-EGFR antibody is ineffective in suppressing compensatory renal hypertrophy [54]. Therefore, 18E1 mAb will not attenuate compensatory renal hypertrophy via EGFR. However, it could reduce through other mechanisms such as inhibition of VEGF signaling [55]. In this study, we also noted that CD148 expression is induced in tubular segments other than collecting ducts in Ang II infused mouse kidneys. Although EGF, TGFβ, and LPS (the ligand for Toll-like receptor 4) were shown to upregulate CD148 in cultured cells [56–59], the mechanism of this is currently unknown. Further investigation would also be required on this subject.

CD148 agonistic antibody may be quite useful for the treatment of kidney disease. First, in the clinical setting, the use of RAS blockade in CKD is often discontinued due to elevation of serum creatinine, hyperkalemia, and hypotension [60, 61]. However, recent studies have shown that the discontinuation of RAS blockade in patients with CKD is associated with a significantly increased risk of all-cause mortality, cardiovascular events, and end stage renal disease (ESRD) [62, 63]. CD148 agonistic antibody may be used in this condition as it would not cause hyperkalemia and hypotension. Second, RAS blockade is the recommended standard therapeutic regimen in CKD. However, in many patients, proteinuria rises again under continued RAS blockade therapy and the aldosterone escape phenomenon was proposed as a mechanism of this [64]. Given the facts that aldosterone upregulates and activates EGFR [15, 16, 65] and EGFR inhibitor effectively suppresses renal injury induced by aldosterone infusion [15], CD148 agonistic antibody may also be used in this setting. Last, a body of literature has shown a critical role of EGFR in the progression of kidney disease [14, 66, 67]. Recently, several EGFR inhibitors have been developed for cancer therapy. However, these EGFR inhibitors showed evident adverse effects in humans, including skin toxicity [14, 17]. This limits their use for CKD because a long term treatment of EGFR inhibitor is expected to cause adverse effects [14]. Since CD148 expression is limited in skin tissue (https://www.genecards.org/cgi-bin/carddisp.pl?gene=PTPRJ), skin toxicity of CD148 agonistic antibody could be much less. Thus, CD148 agonistic antibody may provide an alternative strategy for EGFR inhibition. Further investigation of renal effects of agonistic CD148 antibody may provide another option for the treatment of kidney disease.

## Electronic supplementary material

Below is the link to the electronic supplementary material.

Supplementary Material 1

## Data Availability

The datasets used and analyzed during the present study are available from the corresponding author upon reasonable request.

## Abbreviations

Ang II: Angiotensin II
EGF: Epidermal growth factor
EGFR: Epidermal growth factor receptor
UNx: Unilateral nephrectomy
CKD: Chronic kidney disease
AT1: Angiotensin II receptor type 1
PTP: Protein tyrosine phosphatase
SBP: Systolic blood pressure
ACR: Albumin-to-creatinine ratio
NLS: Nuclear localization sequence
LacZ: β-galactosidase gene
RT: Room temperature
αSMA: Alpha smooth muscle actin
WT1: Wilms’ tumor gene 1
IHC: Immunohistochemistry
p-EGFR: Phospho-EGFR
PDGFRβ: Platelet-derived growth factor receptor-β
CSF1R: Colony-stimulating factor 1 receptor
TGFβR: Transforming growth factor beta receptor
TGFβ: Transforming growth factor beta
ENaC: Epithelial sodium channel
VEGF: Vascular endothelial growth factor
LPS: Lipopolysaccharide
ERK1/2: Extracellular signal-regulated kinase 1/2
PLCγ1: Phospholipase C, gamma 1
