# Supplementary material for: CD148 agonistic antibody alleviates renal injury induced by chronic angiotensin II infusion in mice
Source: BMC Nephrol. 2025 Mar 31;26:165. doi: 10.1186/s12882-025-04070-x (PMC11959730; doi:10.1186/s12882-025-04070-x)
Supplement: Supplementary file 1 — Supplementary Material 1 [file 12882_2025_4070_MOESM1_ESM.pdf]

CD148 agonistic antibody alleviates renal injury induced by chronic angiotensin II infusion in mice

Keiko Takahashi[1], Alina Yu[1], Tadashi Otsuka[1], Lejla Pasic[2], Chikage Narui[1], Lilly He[1], Philipp Ellinger[3], Manuel Grundmann[3], Raymond C. Harris[1], Takamune Takahashi[1]

[1]Division of Nephrology and Hypertension, Vanderbilt University Medical Center, Nashville, Tennessee 37232, USA

[2]Department of Biochemistry, Vanderbilt University, Nashville, Tennessee 37232, USA

[3] Bayer AG Research & Development, Pharmaceuticals, 42113 Wuppertal, Germany

Supplemental Information

Supplemental Methods and References

EGFR Immunohistochemistry

Total EGFR immunohistochemistry was carried out and quantified as follows. Kidney tissues were fixed with 4% paraformaldehyde and paraffin sections were subjected to antigen retrieval with Tris-EDTA pH 9.0 buffer using a pressure cooker for 15 min and cooled to room temperature. Sections were blocked with 0.3% H_2O_2 in methanol followed by the Avidin/Biotin Blocking Kit (Vector Laboratories), then with 1x Universal Blocking Reagent (BioGenex). Tissues were incubated overnight with rabbit anti-EGFR antibody (D1P9C, Cell Signaling Technology) at 4°C, then incubated with biotinylated anti-rabbit IgG (Vector Laboratories) for 1 hr at room temperature. Immunoreactions were detected with the VECTASTAIN Elite ABC HRP Kit and Peroxidase DAB Substrate Kit (Vector Laboratories). Tissues were dehydrated and mounted with Cytoseal XYL (Thermo Fisher Scientific). The results were photographed at 10x magnification by light microscopy (Olympus BX53) and quantified using QuPath version 0.4.3 [1]. Images were annotated to isolate cortex areas, then subjected to a pixel thresholder adjusted to detect DAB

staining positive area for total EGFR expression. The thresholder was run across all image annotations to measure EGFR-positive area per cortex area.

REFERENCES

1. Bankhead P, Loughrey MB, Fernandez JA, Dombrowski Y, McArt DG, Dunne PD, McQuaid S, Gray RT, Murray LJ, Coleman HG *et al*: QuPath: Open source software for digital pathology image analysis. *Sci Rep* 2017, 7(1):16878.

Supplemental Figures

Supplemental Fig. 1 β-Galactosidase histochemistry of CD148 NLS-LacZ knock-in mice that were subjected to UNx alone.

Supplemental Fig. 2 EGFR phosphorylation in glomeruli.

Supplemental Fig. 3 EGFR immunohistochemistry.

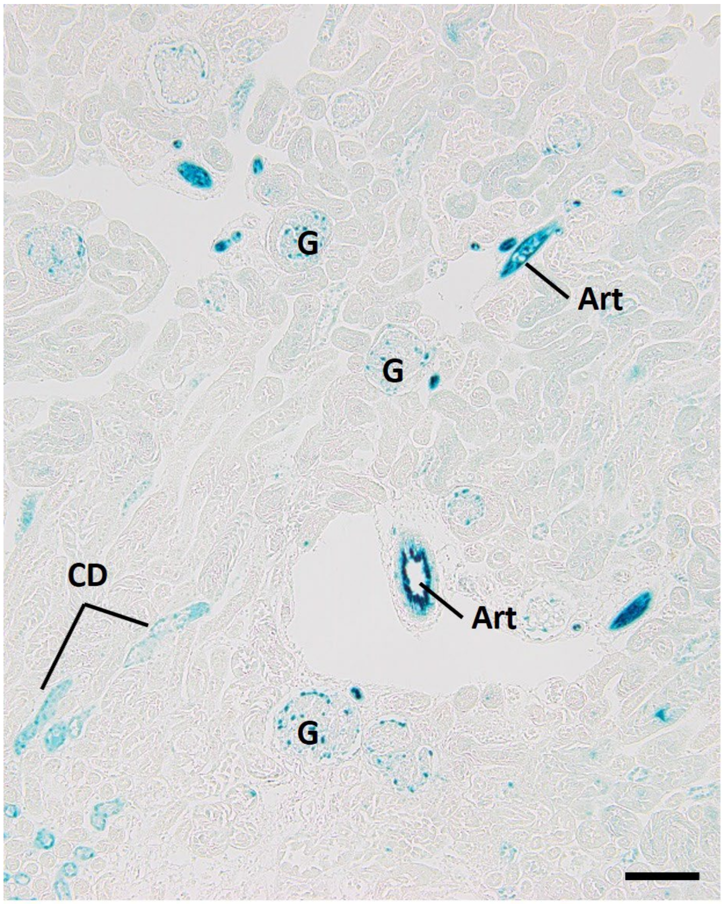

Scale bar: 100 μm

Supplemental Fig. 1 β-Galactosidase histochemistry of CD148 NLS-LacZ knock-in mice that were subjected to UNx alone. Representative data of three mice is shown. G, glomerulus; Art, artery; CD, collecting duct.

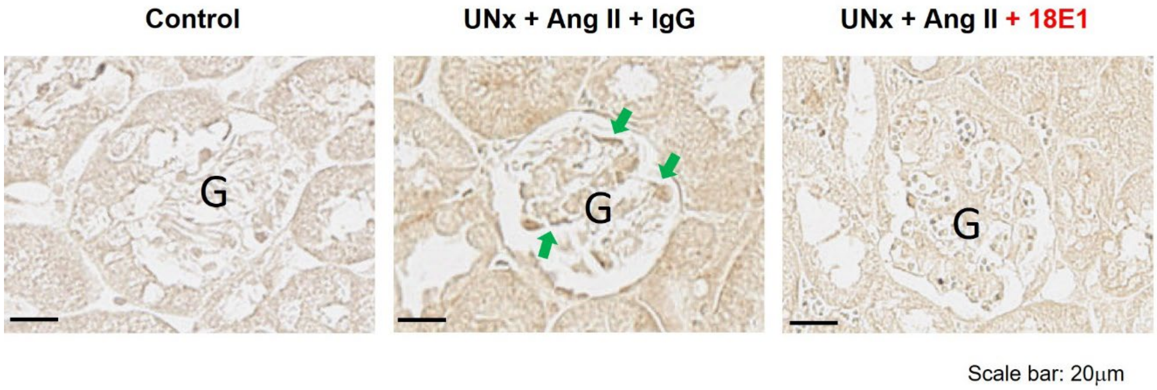

Supplemental Fig. 2 EGFR phosphorylation in glomeruli. EGFR phosphorylation was assessed by immunohistochemistry using the phospho-specific EGFR (Y1068) antibody in each group of mice as in Fig. 4. Photographs show representative results. In UNx + Ang II mice that were treated with a control antibody (IgG), EGFR phosphorylation was observed in podocytes (green arrows) in sclerotic glomerulus. G, glomerulus.

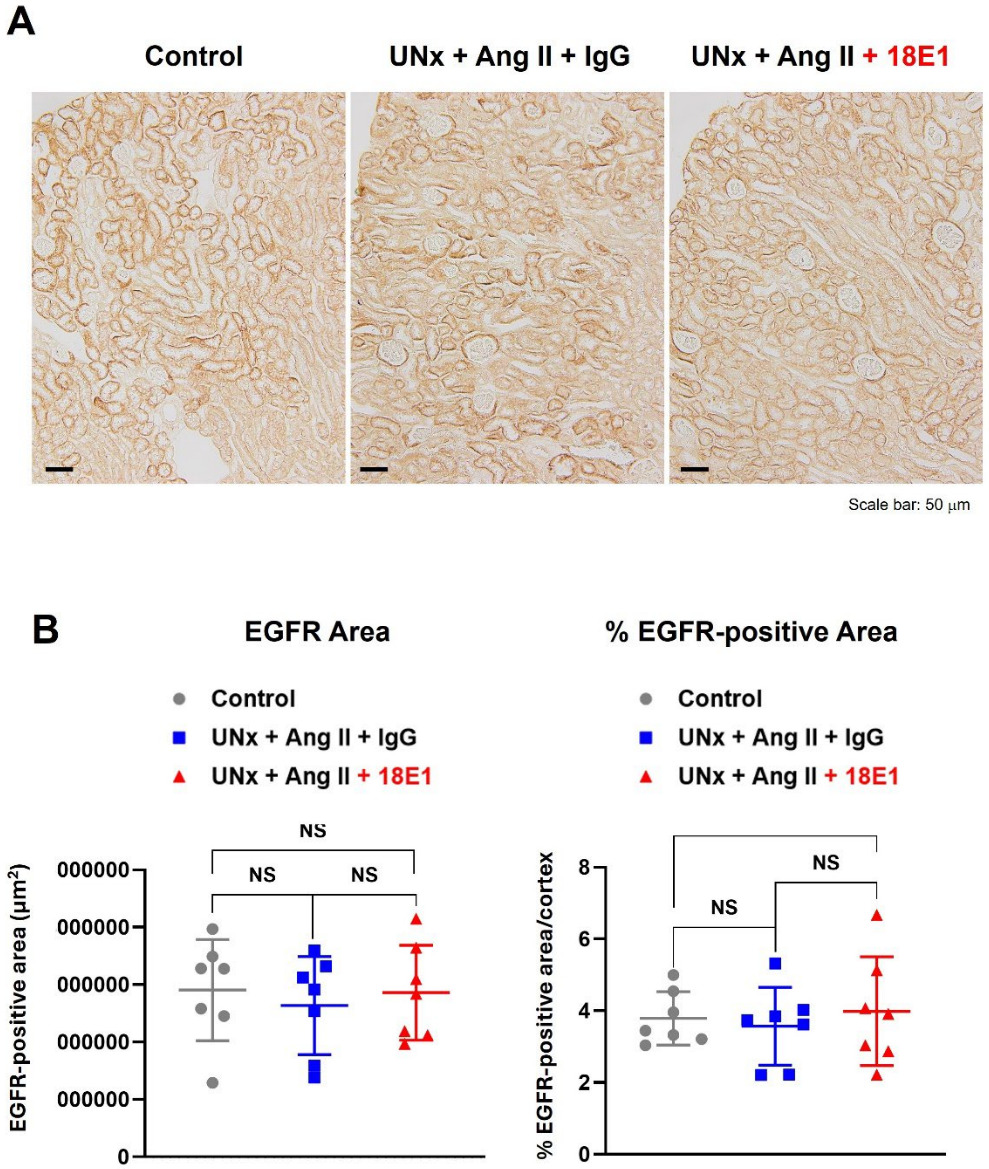

Supplemental Fig. 3 EGFR immunohistochemistry. Total EGFR (EGFR) expression was assessed by immunohistochemistry (A) and quantified (B) as described in the "Supplemental Methods". Photographs show representative results. Data are presented as means ± SD. n=7 for each experimental group.
